# Design of a multi-epitope vaccine candidate against Helicobacter pylori in gastric cancer: an immunoinformatic approach

**DOI:** 10.3205/dgkh000556

**Published:** 2025-06-16

**Authors:** Ali Shojaeian, Samira Sanami, Shahab Mahmoudvand, Razieh Amini, Abbas Alibakhshi

**Affiliations:** 1 Research Center for Molecular Medicine, Institute of Cancer, Avicenna Health Research Institute, Hamadan University of Medical Sciences, Hamadan, Iran; 2 Abnormal Uterine Bleeding Research Center, Semnan University of Medical Sciences, Semnan, Iran; 3 Department of Virology, School of Medicine, Hamadan University of Medical Sciences, Hamadan, Iran

**Keywords:** Helicobacter pylori, multi-epitope vaccine, gastric cancer, molecular docking, molecular dynamics, SabA, BabA

## Abstract

**Background:**

Gastric cancer and peptic ulcers can both be caused by *Helicobacter pylori (H. pylori)*. The complexity of such a bacterium has made it difficult to develop an effective treatment. Thus, a computational approach to developing antigenicity, stability, and safety in vaccines against this pathogen will aid in the management of related diseases.

**Methods:**

This investigation chose two *H. pylori* proteins, SabA and BabA, as epitope prediction targets, and an immunoinformatics platform was used to create a subunit vaccine against* H. pylori*. The best helper T-lymphocyte (HTLs) along with cytotoxic T-lymphocyte (CTLs) epitopes were chosen according to antigenicity, toxicity and allergenicity. The chosen epitopes, suitable linkers, and adjuvants were combined for creating a final vaccine design. The antigenicity, allergenicity, and physicochemical traits of the vaccine were assessed.

**Results:**

The 3D structure of the multi-epitope vaccine was successfully predicted. The results of molecular docking analysis along with molecular dynamics (MD) simulation on the multi-epitope vaccine and immune receptors complex showed the structure has appropriate interaction energy between its two components and good stability. The vaccine candidate was cloned in silico in the pET28a (+) vector successfully in a suitable site.

**Conclusion:**

The results showed that final vaccine design would work well as an effective prophylactic vaccine against *H. pylori*. To evaluate vaccine efficacy against the aforementioned bacteria, *in vivo* and *in vitro* trials are required.

## Zusammenfassung

**Hintergrund:**

Sowohl Magenkrebs als auch Magenulzera können durch *Helicobacter (H.) pylori* verursacht werden. Aufgrund der Komplexität dieses Bakteriums ist es schwierig, eine wirksame Behandlung zu entwickeln. Ein computergestützter Ansatz zur Entwicklung von Antigenität, Stabilität und Sicherheit von Impfstoffen gegen diesen Erreger wird daher bei der Behandlung der damit verbundenen Krankheiten helfen.

**Methode:**

Für die Untersuchung wurden zwei *H. pylori*-Proteine, SabA und BabA, für die Epitopvorhersage ausgewählt. Es wurde eine immuninformatorische Plattform verwendet, um einen Untereinheit-Impfstoff gegen *H. pylori* zu entwickeln. Die besten Epitope für Helfer-T-Lymphozyten (HTLs) und zytotoxische T-Lymphozyten (CTLs) wurden nach Antigenität, Toxizität und Allergenität ausgewählt. Die ausgewählten Epitope, geeignete Links und Adjuvantien wurden kombiniert, um ein endgültiges Impfstoffdesign zu erstellen. Die Antigenität, Allergenität und die physikochemischen Eigenschaften des Impfstoffs wurden bewertet.

**Ergebnisse:**

Die 3D-Struktur des Impfstoffs wurde vorausberechnet. Für den Multiepitop-Impfstoff wurden molekulare Andock-Analysen und Molekulardynamiksimulationen (MD) durchgeführt. Der Impfstoffkandidat wurde in silico in den pET28a (+)-Vektor geklont.

**Schlussfolgerung:**

Der endgültige Impfstoffentwurf ist als wirksamer prophylaktischer Impfstoff gegen *H. pylori* geeignet. Um die Wirksamkeit des Impfstoffs zu bewerten, sind In-vivo- und In-vitro-Versuche erforderlich.

## Background

The most prevalent chronic infectious illness in the world is caused by *Helicobacter (H.) pylori*, which influences around 44.3% of the global population [1]. *H. pylori* infections are more prevalent in less developed nations than in more advanced ones. It was estimated that 54% of Iranians were infected with *H. pylori* [2]. Peptic ulcers and chronic gastritis are just two of the many gastrointestinal disorders linked to *H. pylori* infection. Gastric intestinal metaplasia (GIM) or chronic atrophic gastritis (AG) are two forms of gastritis that were linked to a higher cancer risk [3]. Despite several problems, including the spread of antibiotic resistance, *H. pylori* infections are currently typically treated with a triple antibiotic regimen [4], [5]. 

However, in recent years, antimicrobial resistance to *H. pylori* has increased globally. Recent data from throughout the world shows that the effectiveness of antibiotics used to treat *H. pylori* infections has drastically decreased [6]. Gastric cancer prevention recommendations call for *H. pylori* eradication in population groups with a high risk of contracting the disease [7]. To date, no licensed vaccine candidates against *H. pylori* exist. Therefore, creating a prophylactic vaccine to prevent *H. pylori* infection may be a practical and affordable method of doing so-called epitope-based vaccines, which represent an exciting new approach to creating a distinctive and effective vaccine [8]. These vaccines have piqued the interest of researchers because of their safety, specificity, stability, and low manufacturing cost [9]. Antigen target screening is critical for generating an effective epitope-based vaccine and is essential for vaccine development. In recent years, reverse vaccinology based on bioinformatics has been successfully employed to predict epitopes. 

An epitope is the antigenic portion of a pathogen that is recognized by the host's immune system [10]. Immunoinformatics methods have been created to anticipate the most effective immunogenic epitopes. Immunoinformatics is a precise, reliable, and rapid approach to creating vaccines against pathogens. Until now, several multi-epitope vaccines have been developed against bacteria such as *Escherichia coli *(*E. coli*), *Leptospira* spp., and *Mycobacterium abscessus* [11], [12], [13]. In addition, several epitope-based vaccines against *H. pylori* have been created [14], [15], [16], [17].

Sialic acid-binding adherence (SabA) and blood-group antigen-binding adhesion (BabA) of *H. pylori* have been proposed as attractive options for *H. pylori* vaccine development [18], [19], [20]. BabA and SabA have a vital part in binding *H. pylori* to human gastric tissues, because binding is the first step in *H. pylori* fixation and colonization. As a result, due to the crucial function of BabA and SabA for successful colonization and persistent infection, these antigens can be regarded ideal candidates for developing vaccines [20].

Innate immunity is triggered and the adaptive immune response is synchronized by toll-like receptors (TLRs) [21]. One of the TLRs important in creating immune responses against bacteria is TLR4. Immune cells, e.g., immature dendritic cells (DCs), monocytes, macrophages, as well as granulocytes, express TLR-4 [22]. TLR4-mediated recognition of *H. pylori* LPS was demonstrated for the first time by Kawahara et al. [23]. Given the background mentioned above and regarding the part of *H. pylori* in developing gastric cancer, we aimed to develop a *H. pylori* epitope-based vaccine using the immunoinformatic approach. 

## Methods

Two proteins from *H. pylori*, BabA and SabA, were employed in the present work to predict t-cell epitopes for creating the final vaccine. The epitopes were connected with the vaccine candidate’s design utilizing suitable linkers. To validate the vaccine design, we conducted molecular docking, molecular dynamic simulations, as well as *in silico* cloning. 

### Retrieval of protein sequence 

NCBI (https://www.ncbi.nlm. nih.gov/) provided BabA (NP_045512.2) and SabA (NP_045512.2) amino acid sequences from *H. pylori* in FASTA format.

### Identification and selection of T-cell epitopes 

Using NetCTL 1.2, the target proteins, CTL epitopes were identified (http://www.cbs.dtu.dk/services/NetCTL/). The 12 MHC class I supertypes are the only ones for which such server could anticipate CTL epitopes (9-mer). In the present work, the epitope prediction threshold was established as 0.75. The HTL epitopes were recognized by the NetMHCII 2.3 server (http://www.cbs.dtu.dk/services/NetMHCII/). This server uses artificial neural networks to anticipate how HTL epitopes (15-mer) will interact with HLA-DR, HLA-DP and HLA-DQ. Threshold values of strong and weak binders were established as 2% and 10%, respectively, in this investigation. Antigenicity, toxicity, and allergenicity tests were conducted on them in order to identify the optimal epitopes. Using protein sequences translated to uniform vectors with significant amino acid traits based on auto cross-covariance (ACC), the VaxiJen v2.0 server (http://www.ddg-pharmfac.net/vaxijen/VaxiJen/VaxiJen.html) anticipates epitope antigenicity. In both validations, the model performed well with a prediction accuracy between 70% and 85% [24]. The ToxinPred server (http://crdd.osdd.net/raghava/toxinpred/) was employed to evaluate the toxicity of the anticipated epitopes. Thus, the server predicts essential physical and chemical traits plus toxicity [25]. Using the AllerTOP v.2.0 server, the allergenicity of the predicted epitopes was further evaluated (https://www.ddg-pharmfac.net/AllerTOP/). This technique is supported by the ACC translation of protein sequences to uniform vectors with similar lengths [26].

### Construction of the multi-epitope vaccine 

A multi-epitope vaccine construct was created utilizing HTL and CTL epitopes selected in an earlier step. All of the chosen epitopes were linked using various linkers. Thus, HTL epitopes were joined together utilizing GPGPG linkers, whereas CTL epitopes were connected using AAY linkers. Linkers are employed for minimizing the possibility of producing junctional antigens as well as for enhancing the presentation and processing of antigens [27]. Also, the immunogenicity of multi-epitope vaccines may be improved by using proper linkers [28].

### Evaluation of the antigenicity, allergenicity, and physicochemical properties of the designed vaccine 

To predict the antigenic behavior of the final vaccine design, two servers were used: ANTIGENpro (http://scratch.proteomics.ics.uci.edu/) as well as VaxiJen v2.0. Furthermore, the AllerTOP v. 2.0 and ToxinPred servers were employed to evaluate the allergenicity and toxicitiy of the vaccine construct, respectively. The Expasy Protpram server (https://web.expasy.org/protparam/) was employed to characterize several physicochemical characteristics, such as molecular weight, theoretical pI, instability index, aliphatic index, as well as grand average of hydropathy (GRAVY).

### Prediction of the secondary structure

The Prabi server (https://npsa-prabi.ibcp.fr/cgi-bin/npsa_automat.pl) was utilized to anticipate the vaccine construct’s secondary structure. This server predicts a secondary structure using the GOR IV approach with a 64.4% average accuracy [29].

### Tertiary structure modeling, refinement, and validation of the multi-epitope vaccine 

The I-TASSER server (https://zhanglab.ccmb.med.umich.edu/I-TASSER/) was utilized to generate the vaccine construct’s final three-dimensional model. Thus, I-TASSER is a system for generating accurate models of protein tertiary structures from their amino acid sequences. This server reconstructs segments clipped from threading templates to produce 3D models based on an amino acid sequence, and it then assigns a C-score to each model to indicate its level of quality [30]. After that, the protein structural-refinement server 3D-refine server (http://sysbio.rnet.missouri.edu/3Drefine/) was used. The 3D-refine methodology performs the iterative optimization of the hydrogen bonding network, as well as atomic-level energy reduction on the optimized model in order to effectively enhance protein structures [31]. Both the ProSA-web server (https://prosa.services.came.sbg.ac.at/prosa.php) and the SAVES v6.0 server (https://saves.mbi.ucla.edu/) were used to validate the models. Based on the total quality of the protein model, the ProSA server determined z-scores for each protein model. Any structure with a Z-score beyond the typical range is likely to be flawed [32]. The SAVES v6.0 server analyzes the geometry of residues as well as the total structural geometry to rate the stereochemical quality of a protein structure [33].

### Molecular docking 

ClusPro 2.0 server (https://cluspro.org/login.php) has been utilized for assessing the contact among the TLR4 and vaccine construct (PDB ID: 4G8A). This server is a docking server for two interacting proteins. The ClusPro server, which is frequently used for the docking homology model, constructs structures using three separate coefficient sets [34].

### Molecular dynamics simulation 

GROMACS 5.1.1 software and GROMOS96 54a7 force field were used for molecular dynamics simulation (MD). The GROMACS program forecasts ligand and receptor behavior over time utilizing Newton’s equations of atomic and molecular motion [35], [36]. Using the SPC/E water model, MD simulations were performed between TLR4 and the vaccine construct, as well as the complex of the TLR4-vaccine construct. Van der Waals interactions and hydrogen bonds that developed among complex and water molecules were deleted during the energy minimization of the structures using the steepest descent methodology. Thereafter, the temperature was gradually increased from 0 to 300 K, bringing the system to equilibrium at constant pressure, with both phases at 100 ps, in a constant volume. The MD simulation took place for 30 ns at a temperature of 300 K. The root mean square fluctuation (RMSF), root mean square deviation (RMSD) and radius of gyration (Rg) of the ligand and receptor complex were then determined.

### Codon optimization and in silico cloning of the final vaccine construct 

For codon optimization along with reverse translation of vaccine components, the Java Codon Adaptation Tool (JCat) (http://www.jcat.de/) was employed [37]. The the codon adaptation index (CAI) along with GC content are two parameters that influence protein expression. An increased chance of protein expression is indicated by a CAI value >0.8. Any gene should have a GC content of between 30% and 70% to produce proteins effectively [38], [39]. In the present work, the vaccine construct’s main sequence was enhanced by using *E. coli* strain K12 as the host organism. Finally, using the restriction enzymes *BamHI* and *XhoI*, the optimized codon sequence was cloned to the pET28a (+) vector utilizing the SnapGene program.

## Results

### Selected T-cell epitope 

Utilizing the NetCTL 1.2 server, sixty CTL epitopes were predicted for the BabA and SabA proteins. The anticipated epitopes underwent several tests. The initial set of epitopes chosen can bind to three or more MHC class I supertypes. The antigenicity, allergenicity, and toxicity of these epitopes were then assessed utilizing the VaxiJen v2.0, AllerTOP v2.0 and ToxinPred v2.0 servers. Finally, a CTL epitope was determined for both BabA and SabA (Table 1 (Tab. 1)). Here, 90 HTL epitopes for BabA and SabA proteins were predicted using the NetMHCII 2.3 server, and 17 epitopes that might attach to a minimum of 3 MHC class-II alleles were examined for antigenicity, toxicity, and allergenicity. As a result of these tests, we were able to narrow down the pool of potential HTL epitopes to just two for BabA and three for SabA (Table 2 (Tab. 2)). 

### The multi-epitope vaccine construct 

**Figure 1:**
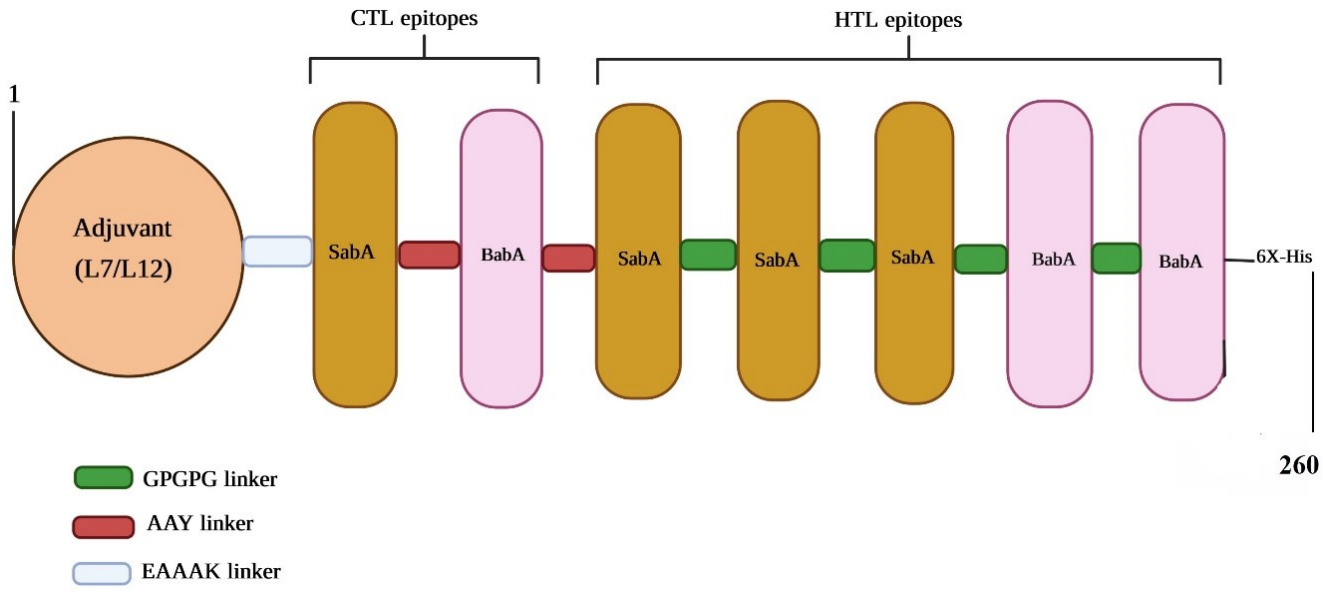
An illustration of the multi-epitope vaccine’s structural organization

By other resaercher a multi-epitope vaccine design has been frequently produced by mixing two CTL epitopes along with five HTL epitopes, utilizing the GPGPG and AAY linkers, respectively. When epitopes and linkers were fused, a 130-amino-acid sequence resulted. Then, we opted for the L7/L12 protein from the 50S ribosomal subunit (Accession no. P9WHE3) to boost immunogenicity. After that, epitopes for CTLs and HTLs were included. To complete the purification process, the C-terminal region received a 6x-His tag (Figure 1 (Fig. 1)).

### Evaluation of the physicochemical properties, antigenicity, allergenicity, and solubility of the construct 

**Figure 2:**
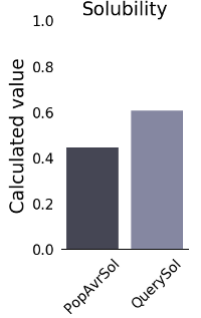
Solubility diagram of multi-epitope vaccine calculated via Protein-Sol server

The physicochemical traits of the designed vaccine were determined using the ProtParam server (Table 3 (Tab. 3)). The ultimate multi-epitope vaccine has a total of 260 amino acids. The vaccine has a GRAVY score of –0.385, which indicates how hydropathic it is, and an aliphatic index of 65.15. High aliphatic index proteins are more durable across a wider temperature range. The multi-epitope vaccine has a total of 294 amino acids. Thus, the theoretical pI and molecular mass of the vaccine design were found to be 6.25 and 26.14 kDa, respectively. It has been determined that the instability index is 17.09. This means that the protein is considered stable. Heterologous expression within bacteria and yeast requires a long half-life. The vaccines’ half-lives in mammalian reticulocytes *in vitro*, yeast and *E. coli in vivo* were determined to be 30 hours, 20 hours, and 10 hours, respectively. The vaccine design’s antigenicity was assessed utilizing the servers ANTIGENpro and VaxiJen v2.0. VaxiJen v2.0 and ANTIGENpro estimated the antigenicity to be 1.0721 and 0.941287, respectively. The AllerTOP v. 2.0 server was employed to test the recommended vaccine for allergenicity, and the findings revealed that it was hypoallergenic. The SOLpro server predicted solubility while overexpressed in *E. coli* with a very high degree of accuracy (0.968397). A projected scaled solubility value of 0.609 was likewise reported by the Protein-sol service (Figure 2 (Fig. 2)). Scaled solubility values over 0.45 are anticipated to possess better solubility in comparison to the typical experimentally soluble *E. coli* protein, while scaled solubility values below 0.45 are predicted to possess less solubility. Thus, the experimental data set’s PopAvrSol population average is 0.45.

### Secondary and tertiary structures, refinement, and validation of the construct

**Figure 3:**
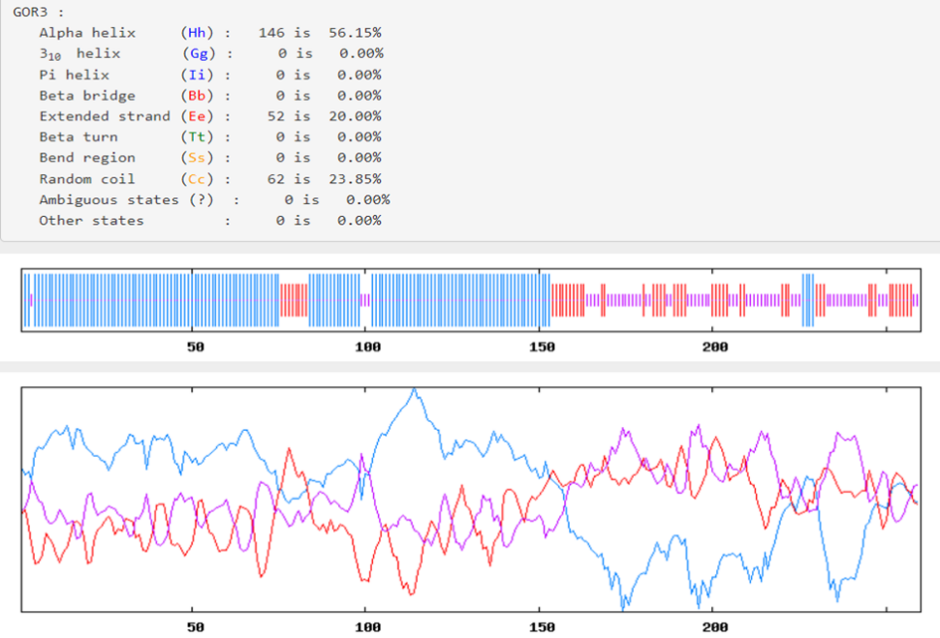
Graphic depiction of the secondary structure of the multi-epitope

**Figure 4:**
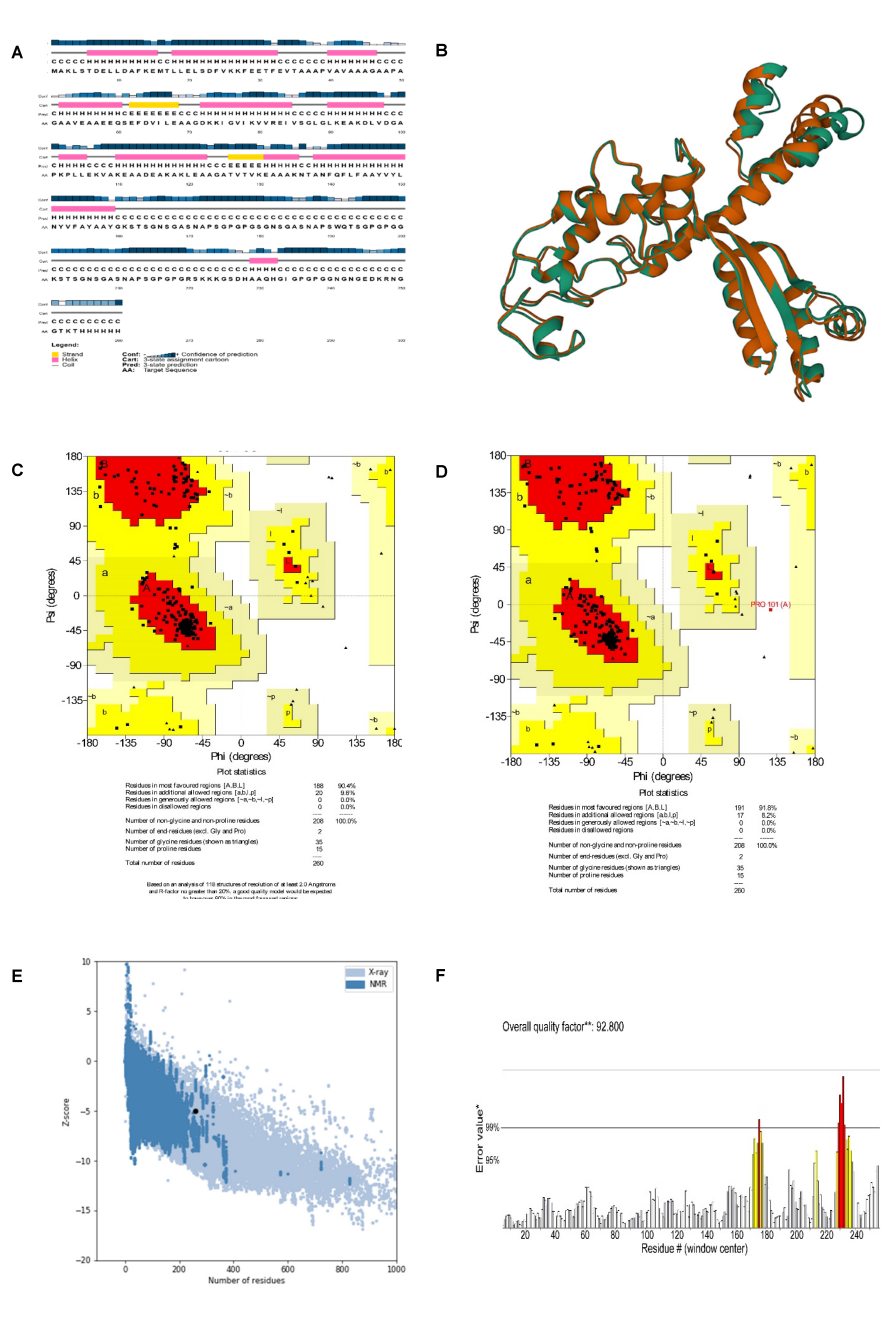
Table 4: Outcomes of the model’s refinement. Models of better quality have lower RWplus and MolProbity values and stronger GDT-TS, GDT-HA, and RMSD values

The Prabi server was employed to determine the secondary structure components’ percentage in the multi-epitope vaccine. Extended strand (20%), random coil (23.85%), and alpha-helix (56.15%) were all predicted structures (Figure 3 (Fig. 3)). The 3-dimensional structure of vaccine construct was modeled five times by the Robetta server. The model having the highest C-score out of five was picked. The higher C-score for the model denotes a high degree of confidence, with the C-score often falling between 5 and 2 [40]. As a result, we chose model 1. The Chimera 1.15rc software was used to visualize 3D vaccine construction models [41] (Figure 4 (Fig. 4)). The 3D-refine server then refined the model. The 3D-refined score, GDT-TS, RMSD, GDTHA, RWPlus and MolProbity were all provided by this server with varied parameters (Table 4 (Tab. 4)). Better model quality is indicated by stronger GDT-TS, RMSD and GDT-HA values and weaker 3D-refine scores, MolProbity and RWplus values. Based on the factors listed above, refined model 5 was chosen (Figure 4 (Fig. 4)). We also compared the total quality of the multi-epitope vaccine’s protein structure before and after the refinement using the ProSA-web and SAVES v6.0 servers. Then, the modified model’s Z-score was –4.96 (Figure 4 (Fig. 4)). The total quality of the multi-epitope vaccine’s protein structure was compared before and after the refining procedure using the ProSA-web and SAVES v6.0 servers. Thus, the Z-score for the improved model was –4.96 (Figure 4 (Fig. 4)). The Ramachandran plot produced via the SAVES v6.0 server shows that the first model had 90.4%, 9.6%, 0.0%, and 0.0% of the residues present in the favored, additional allowed, generously allowed, and disallowed regions, respectively (Figure 4 (Fig. 4)), whereas the refined model had 91.8%, 8.2%, 0.0%, and 0.0% of the residues present, respectively (Figure 3 (Fig. 3)).

### Molecular docking 

**Figure 5:**
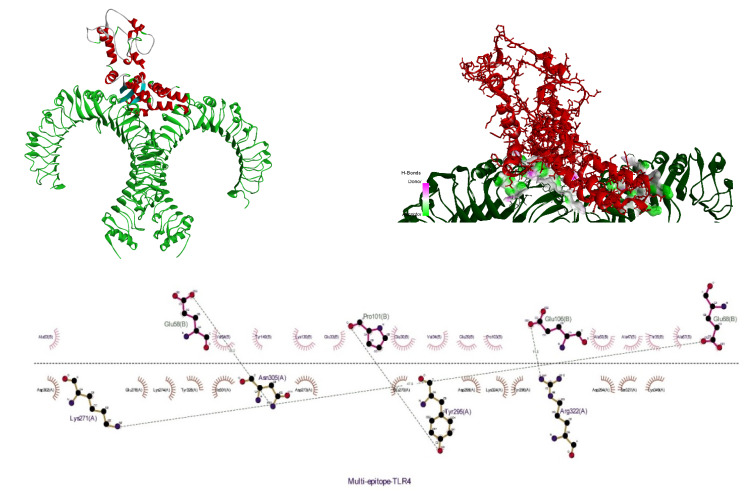
3D and 2D diagram of docked complexes of multi-epitope structure and immune receptors – Multi-epitope TLR4

**Figure 6:**
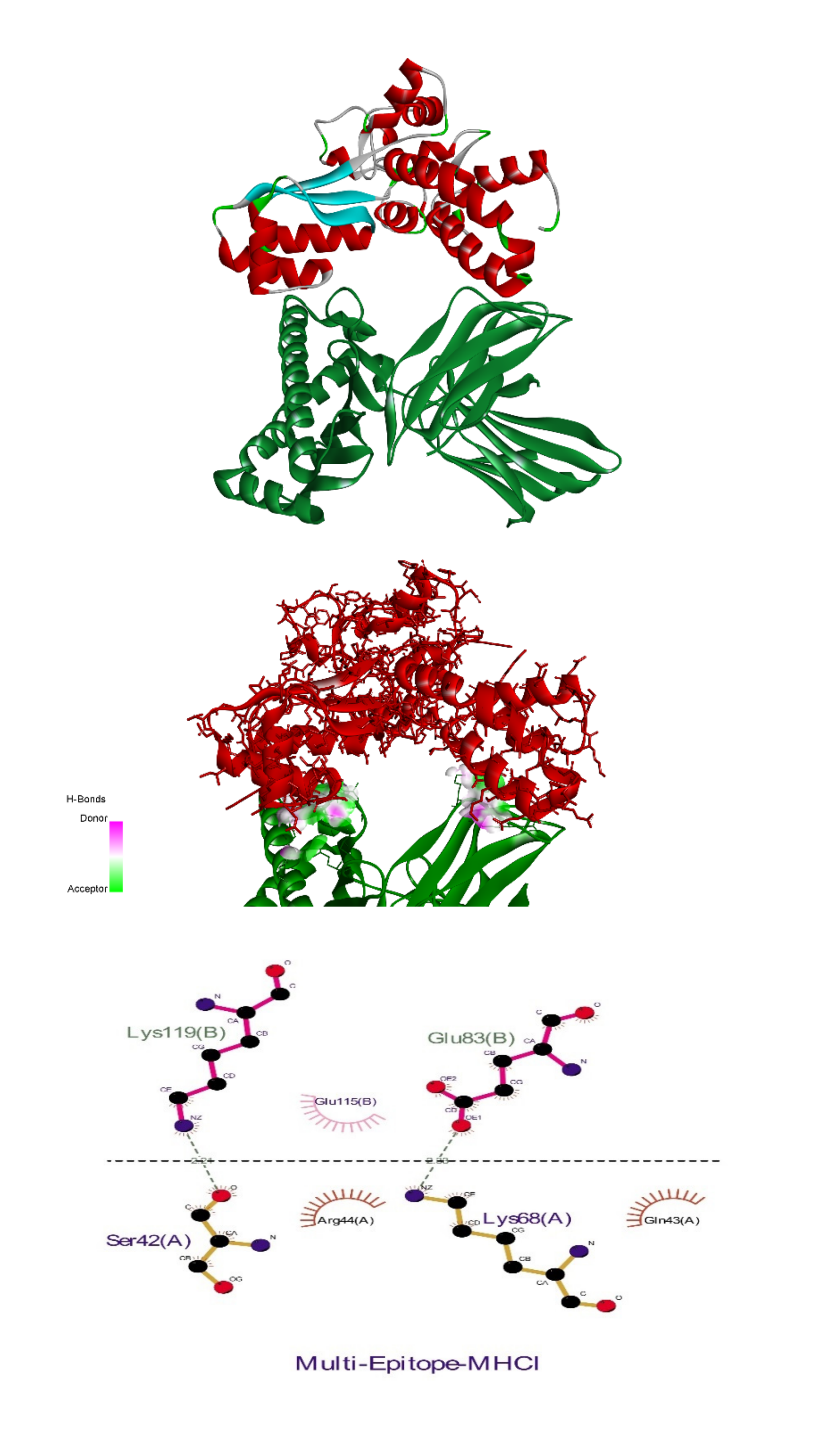
3D and 2D diagram of docked complexes of multi-epitope structure and immune receptors – Multi-epitope MHCI

**Figure 7:**
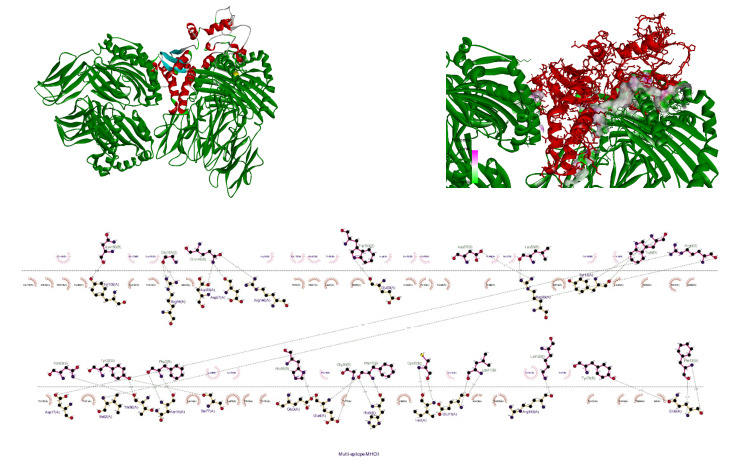
3D and 2D diagram of docked complexes of multi-epitope structure and immune receptors – Multi-epitope MHCII

The receptor and ligand were docked to each other after the preparation process, then the best complex was refined. The degree of binding and the strength of the interaction between the two components are defined by the binding energy, which in this study showed the values of –11.78, –13.15, and –42.91 for the binding of multi-epitope to all three receptors, TLR4, MHCI, and MHCII, respectively. Here, these binding affinities were scored based on different energies, including van der Waals, partial electrostatic, aliphatic, and other strong bonds. Then, the binding of this epitope to the receptors was checked schematically (Figure 5 (Fig. 5), Figure 6 (Fig. 6), and Figure 7 (Fig. 7)) and then the protein-protein docking was checked in terms of amino acid involvement. Figure 5 (Fig. 5), Figure 6 (Fig. 6), and Figure 7 (Fig. 7) show that significant amino acid involvement exists for two immune system receptors, TLR4 and MHCII.

### MD simulation 

**Figure 8:**
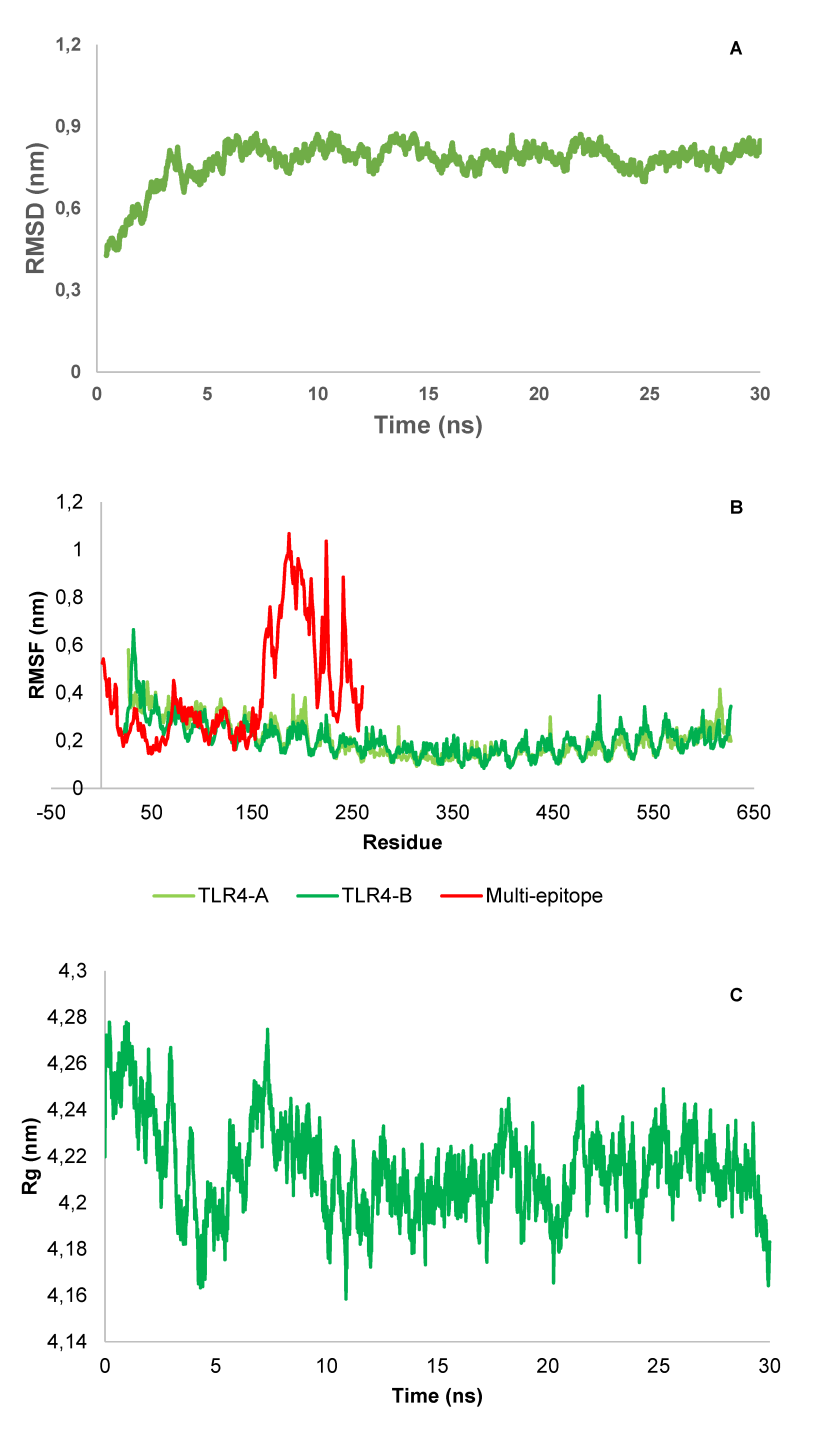
RMSD (A), RMSF (B), and radius of gyration (C) of the multi-epitope as well as immune receptor (TLR4) complex

The MD simulation revealed the function of the studied protein construct, the interactions involved, and the protein structure’s stability. The result analysis of the multi-epitope and immune receptor complex studied here showed that the docked construct has relative stability after minimizing energy and reaching equilibrium. The RMSD plot shows that the docked structure stabilized after approximately 5 ns (Figure 8A (Fig. 8)). Also, the average of the last 5 ns shows the value of 0.791±0.023. The RMSF plot that shows atomic fluctuations representing an MD for 30 ns of both multiepitope and TLR-4 demonstrated that the binding of multiepitope to TLR-4 resulted in a decreased flexibility of the residues and a relative stability (Figure 8B (Fig. 8)). Moreover, for further analysis, the Rg plot complex after an MD 30 ns was also drawn (Figure 8C (Fig. 8)) and showed an average value of 4.213±0.014 for the last 5 ns, which together show relatively high compactness for this complex.

### In silico cloning 

**Figure 9:**
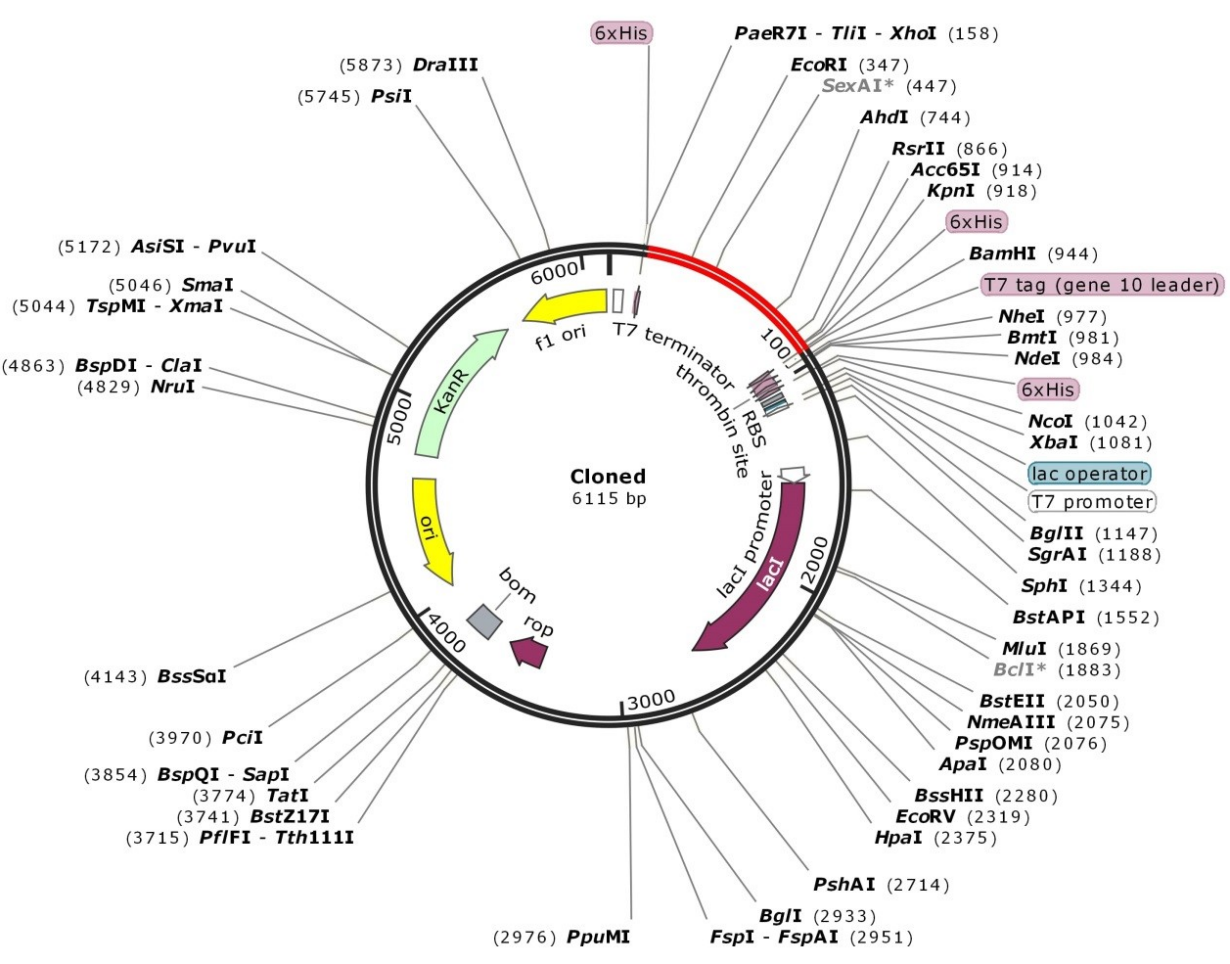
SnapGene software (https:// www. snapgene. com/free- trial/) in silico cloning map of the multi-epitope vaccine into the pET28a (+) vector. The red arc is the vaccine’s structure, and the black arc is the backbone of the vector.

The multi-epitope vaccine’s codon optimization and reverse translation were conducted by JCat. The CAI of the improved vaccine nucleotide sequence is 1.00, and its GC content is 51.28%. The vaccine design was then virtually cloned in the pET-28 (+) vector utilizing the SnapGene program (Figure 9 (Fig. 9)).

## Discussion

Infection with *H. pylori* has been related to a variety of gastrointestinal disorders, including peptic ulcers and chronic gastritis. Both cancer and precancerous lesions, e.g., chronic atrophic gastritis (AG) or gastric intestinal metaplasia (GIM), have been linked to it [3]. To prevent issues with antibiotic therapy for* H. pylori* infection (recurrence, increasing resistance, flora disruptions, etc.), vaccines may be a preferable choice, also due to their safety and effectiveness [42]. Conventional techniques of vaccine development are now all but obsolete due to their poor effectiveness and high financial and labor costs. Many scientists throughout the world are interested in reverse vaccinology, a method for creating new vaccines that merges immunogenicity and bioinformatics [43]. An immunoinformatic approach to vaccine design is more efficient, specific, stable, and reasonably safe. Multi-epitope vaccines have been an effective strategy for complicated pathogens. This approach is commonly utilized to create vaccines against different pathogens, such as *H. pylori* [44], *hepatitis C* virus [45], *Elizabethkingia anopheles* [46], *Fasciola gigantica* [47], *Candida auris* [48], *Tropheryma whipplei* [49], *Leishmania donovani* [50], Zika virus [51], Dengue virus [52], *Klebsiella pneumonia* [53], and SARS-COV-2 [54], [55]. Several studies concentrating on the development of *H. pylori* multi-epitope vaccines have been published recently [9], [56], [57]. According to Meza et al. [56], four pathogenic proteins (FliD, Urease B, VacA, and CagA) have both T- and B-cell epitopes that could be utilized for creating a multi-epitope vaccine against *H. pylori*. Additionally, Khan et al. [58] anticipated T-cell and B-cell epitopes from many pathogenic proteins (CagA, GroEL, OipA, and VacA) for making a multi-epitope vaccine against *H. pylori* [58]. Therefore, we employed immunoinformatic techniques for creating a multi-epitope vaccine against *H. pylori* infection. Two *H. pylori* proteins, BabA and SabA, were used in the current investigation to anticipate T-cell epitopes for the final vaccine formulation.

In this study, two major immuno-protective antigens, BabA and SabA, of *H. pylori* were selected. Because BabA and SabA exhibit strong antigenic properties which are two outer membrane proteins, they are used as a potential candidate in vaccine development against *H. pylori*. SabA interacts with sialylated Lewis antigens, which are crucial throughout the persistent infection phase, whereas BabA interacts with host Lewis antigens during the early infection phase [59]. Furthermore, it has been demonstrated that the presence of BabA is correlated with heightened inflammation of the gastric mucosa and an elevated risk of developing clinical consequences [60].

Intestinal metaplasia, atrophic gastritis, and gastric cancer have also been linked to the SabA antigen [61]. This makes it a good option for use as an adjuvant. For the same reason, these proteins are also a good option for the creation of a vaccine against *H. pylori*.

A crucial stage in the creation of multi-epitope vaccines is the precise detection of epitopes [62]. In this study, we used the NetCTL 1.2 and NetMHCII 2.3 servers to identify CTL and HTL epitopes, respectively. Epitope screening for antigenicity, toxicity, and allergenicity was conducted in order to choose the best epitopes. BabA and SabA proteins were projected to have a combined total of 60 CTL epitopes, and those that might attach to a minimum of three MHC class I supertypes were selected. According to the results of antigenicity, toxicity, and allergenicity, two CTL epitopes VYLNYVFAY (BabA) and NTANFQFLF (SabA) were suitable for designing a multi-epitope vaccine. However, 90 HTL epitopes were predicted, and 17 of these were found to be able to attach to a minimum of 3 MHC class II alleles. Based on results of antigenicity, toxicity, and allergenicity, five HTL epitopes including two epitopes for BabA (RSKKKGSDHAAQHGI, GNGNGEDKRNGGTKT) and three epitopes for SabA (GKSTSGNSGASNAPS, SGNSGASNAPSWQTS, GKSTSGNSGASNAPS) were selected to design the multi-epitope vaccine.

It has been demonstrated that components such adjuvant and linker can have an impact on a multi-epitope vaccine’s ability to successfully elicit the appropriate immune reaction. Relevant linkers were employed in this study to link epitopes and fuse those epitopes with other elements. The primary benefits of employing linkers are improved antigen processing, presentation, and immunogenicity [27]. In reality, two factors that might impact a protein’s immunogenicity are the epitope location and the use of an appropriate linker [28], [63]. In the current work, linkers EAAAK, GPGPG, and AAY were employed to bind several vaccine components together. To bind the 50S ribosomal protein L7/L12 to CTL and HTL epitopes, linker “EAAAK” was used. 

EAAAK is a stiff linker, which leads to a fixed distance between protein domains to maintain their independent function [64]. The use of “GPGPG” to link HTL epitopes has the dual benefits of preventing the development of junctional epitopes and inducing HTL responses [65]. The “AAY” linker functions as a cleavage site for proteasomes within mammalian cells, which lowers junctional immunogenicity. As a result, CTL epitopes were linked together utilizing this linker [63].

Vaccines having several epitopes are frequently immunogenic and must be combined with adjuvants. Adjuvants are an important factor in vaccine development, as they enhance the immunological properties of vaccine constructs. In this work, 50S ribosomal protein L7/L12 was utilized as an adjuvant, which indeed improved potential receptor interactions. The 50S ribosomal protein L7/L12 is a hybrid of the L7 and L12 components. This protein functions as a TLR4 agonist and leads to induced strong responses of Ag-specific CD8+ class I CTL. For this reason, it is a good option for use as an adjuvant [66]. 

Next, the antigenicity, toxicity, allergenicity, and physicochemical characteristics of the vaccine designs were examined. The results of the antigenicity evaluation utilizing the two web servers VaxiJen v2.0 and ANTIGENpro showed that the antigenicity scores for the vaccine design were 0.941287 and 1.0720, respectively. The allergenicity and toxicity data showed that the vaccine design was non-allergic and non-toxic, with a molecular weight of 26.14 kDa. It is easier to purify proteins with molecular weights under 110 kDa [67]. The protein’s theoretical pI was 6.25, and its aliphatic index was 65.15. The GRAVY score was given as –0.385. The hydrophilic character of the vaccine is reflected in the negative GRAVY score, while a stronger aliphatic index value suggests enhanced thermal stability [68]. The calculated instability index is 17.09. It was determined that proteins having an instability score <40 were stable [69]. Furthermore, the solubility analysis performed by the SOLpro server produced a solubility score 0.9683 points higher than the server’s probability of ≥0.5. Also, using the Protein-sol server, the scaled solubility value (QuerySol), which was anticipated to be 0.609, was determined. Scaled solubility values over 0.45 are expected from the experimental solubility to be greater than the average solubility of *E. coli* protein, as the population average in the experimental dataset (PopAvrSol) was 0.45. In mammalian reticulocytes cultured in vitro, in yeast, and in *E. coli* grown in vivo, the vaccine’s half-life was 30 hours, >20 hours, and >10 hours, respectively. Alpha-helix (56.15%), extended strand (20%), and random coil (23.85) were among the expected structural elements, according to the study of the secondary structure performed using the Prabi server. The ProSA Z-score and the Ramachandran plot were used in the current work to assess the initial and improved models’ quality. Previous research revealed that more than 90% of the residues should be found in the plots’ most favored locations [70]. This investigation’s outcomes showed more than 90% of the residues fell inside the targeted area, thus demonstrating the high quality of the suggested model. In the initial model, 90.4%, 9.6%, 0.0%, and 0.0% of the residues existed in the preferred, additional permitted, generously allowed, and prohibited areas, respectively; in the improved model, these percentages changed to 91.8%, 8.2%, 0.0%, and 0.0%. This was demonstrated by a Ramachandran plot. Furthermore, the refined model’s Z-score was –4.96. To be effective, a vaccine design depends on understanding the target protein’s tertiary and secondary structures. The planned vaccine’s 3D structure improved significantly following all refinement stages.

The values –11.78, –13.15, and –42.91 for the binding of the vaccine construct to each of the three TLR4, MHCI, and MHCII receptors, respectively, was determined by docking analysis of the molecular contact of the vaccine construct with TLR4, MHCI, and MHCII. This result indicated that the recommended vaccine interacted more strongly with MHCII than it did with MHCI and TLR4. The outcomes of the molecular simulation demonstrated that the docked protein structure eventually attained relative stability. RMSD was employed to identify large changes in protein structure in the current study; the MD simulation trajectory’s RMSD analysis indicated that the docked complex equilibrated and did not deviate from the original structure in a relatively stable way with time. For further confirmation, these results can also be deduced from the radius of gyration, as a non-significant standard deviation occurred in the MD trajectories for the final nanoseconds. Also, the RMSF plot showed that the relative stability and small deviations can be a reason for the interactions between the two parts of the complex.

Protein expression is influenced by a number of components, such as GC and CAI content. Any gene’s codon expression level may be measured by the CAI, and a CAI value above 0.8 denotes a stronger expression level. In reality, to achieve high-level protein production, codon optimization often increases transcriptional and translational efficiency. Additionally, the GC content should be anywhere from 30% to 70%t to increase the degree of protein expression [38], [39]. The GC and CAI contents in this work were 1.00 and 51.28%, respectively. Lastly, in silico cloning of the optimized sequence was performed into the pET28a (+) vector utilizing SnapGene. This vector is an excellent means of producing vast quantities of protein [71]. Furthermore, the inclusion of the 6 His tag enabled protein separation for later analysis [72]. 

### Limitations

This study relied entirely on computational and *in silico* methods for vaccine design, which presents several limitations. Firstly, while immunoinformatics approaches can predict epitope behavior, they cannot fully replicate the complex biological interactions that occur in living systems, potentially missing important immunological factors. Secondly, the computational predictions of antigenicity, allergenicity, and physicochemical properties, while valuable, require extensive experimental validation, since in silico models cannot perfectly simulate real-world immune responses.

## Conclusions

A prophylactic vaccine against *H. pylori* infection may prove to be practical and affordable. In the current work, we made a multi-epitope vaccine against *H. pylori* utilizing immunoinformatics. The proposed vaccine might be a good vaccine candidate against *H. pylori*, according to in silico research. Additional *in vivo*, preclinical and clinical studies are needed to evaluate the safety along with the effectiveness of the suggested vaccines.

## Notes

### Competing interests

The authors declare that they have no competing interests.

### Funding

This work is a part of research project and was financially supported by Deputy of Research, Hamadan University of Medical Sciences (grant number: 140105183701). 

### Ethics approval

This study was approved by the ethics committee of Hamadan University of Medical Sciences (IR.UMSHA.REC.1401.400).

### Authors’ ORCIDs

- Shojaeian A: https://orcid.org/0000-0002-1166-385X
- Sanami S: https://orcid.org/0009-0008-1050-7880
- Mahmoudvand S: https://orcid.org/0000-0002-9155-9939
- Amini R: https://orcid.org/0000-0002-7588-3552
- Alibakhshi A: https://orcid.org/0000-0003-0402-6892

## Figures and Tables

**Table 1:**
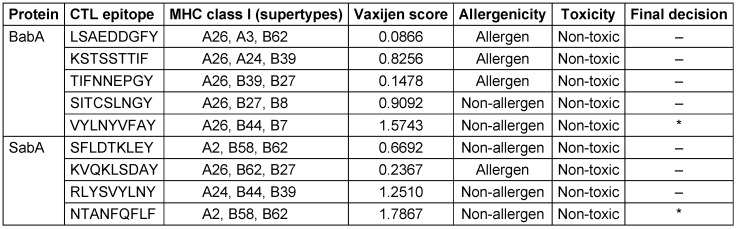
Anticipated CTL epitopes of BabA and SabA proteins

**Table 2:**
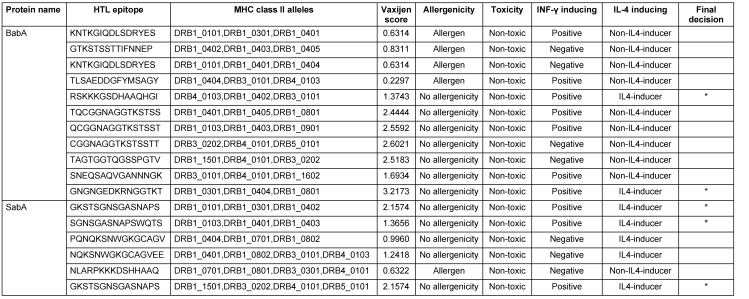
Anticipated HTL epitopes of BabA and SabA proteins

**Table 3:**
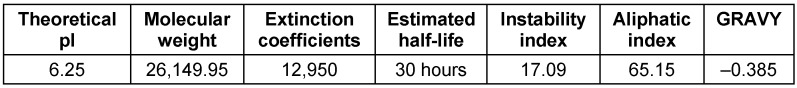
The ProtParam server was used to determine a number of the designed vaccine’s physicochemical traits.

**Table 4:**
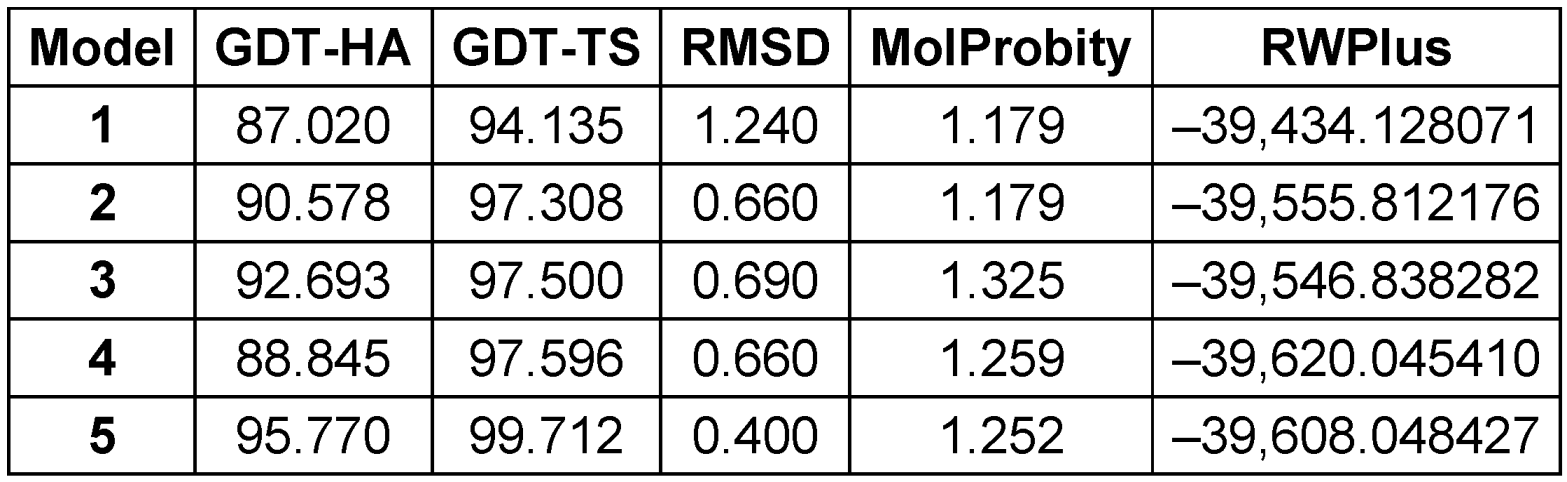
Outcomes of the model's refinement. Models of better quality have lower RWplus and MolProbity values and stronger GDT-TS, GDT-HA, and RMSD values
